# Newly diagnosed with inflammatory arthritis (NISMA)–development of a complex self-management intervention

**DOI:** 10.1186/s12913-022-09007-w

**Published:** 2023-02-07

**Authors:** L. H. Lindgren, T. Thomsen, A. de Thurah, M. Aadahl, M. L. Hetland, S. D. Kristensen, B. A. Esbensen

**Affiliations:** 1 Copenhagen Center for Arthritis Research, Center for Rheumatology and Spine Diseases, Rigshospitalet, Valdemar Hansens Vej 17, 2600 Glostrup, Denmark; 2 Center for Clinical Research and Prevention, Bispebjerg and Frederiksberg Hospital, Copenhagen, Denmark; 3 Department of Rheumatology, Aarhus University Hospital, Aarhus, Denmark; 4 Department of Clinical Medicine, Aarhus University, Aarhus, Denmark; 5 Department of Clinical Medicine, Faculty of Health and Medical Sciences, University of Copenhagen, Copenhagen, Denmark; 6 Patient Research Partner, Copenhagen, Denmark

**Keywords:** Self-management, Inflammatory arthritis, Complex interventions, Development, Newly diagnosed and multi-disciplinary intervention

## Abstract

**Background:**

Patients newly diagnosed with inflammatory arthritis (IA) request regular consultations and support from health professionals to manage physiological, emotional, and social challenges. Evidence suggests that providing a tailored multi-component self-management program may benefit disease management. However, there is a lack of evidence of effective interventions with multiple components targeting the needs of this group. Therefore, the aim of this study was to develop a self-management intervention targeting newly diagnosed patients with IA, following the Medical Research Council (MRC) framework for developing complex interventions.

**Methods:**

The development of the complex self-management intervention covered three steps. First, the evidence base was identified through literature reviews, in which we described a preliminary nurse-led intervention. Secondly, we chose Social Cognitive Theory as the underlying theory along with Acceptance and Commitment Theory to support our communication strategy. Thirdly, the preliminary intervention was discussed and further developed in workshops to ensure that the intervention was in accordance with patients’ needs and feasible in clinical practice.

**Results:**

The developed intervention comprises a 9-month nurse-led intervention (four individual and two group sessions). A physiotherapist and an occupational therapist will attend the group sessions along with the nurse. All sessions should target IA-specific self-management with a particular focus on medical, role, and emotional management.

**Conclusion:**

Through the workshops, we involved all levels of the organization to optimize the intervention, but also to create ownership and commitment, and to identify barriers and shortcomings of the preliminary intervention. As a result, from the existing evidence, we believe that we have identified effective mechanisms to increase self-management in people newly diagnosed with IA. Further, we believe that the involvement of various stakeholders has contributed significantly to developing a relevant and feasible intervention. The intervention is a nurse-led complex self-management intervention embedded in a multidisciplinary team (named NISMA). The intervention is currently being tested in a feasibility study.

**Supplementary Information:**

The online version contains supplementary material available at 10.1186/s12913-022-09007-w.

## Background

Inflammatory arthritis (IA) covers a group of diseases caused by an overactive immune system. The most common types of IA are Rheumatoid Arthritis (RA), axial Spondyloarthritis (axSpA), and Psoriatic Arthritis (PsA) [1]. These three types of IA affect more than 2% of the population worldwide, with considerable variation among ethnicities [2–4]. In Denmark, approximately 80,000 suffers from IA, with RA being the most common [5], and the socioeconomic costs of RA in Denmark are estimated at 24.000 US dollars per person per year [6].

IA can occur at any age and in both sexes. The cause of IA is multifactual and involves both genetic and lifestyle factors [4, 7]. IA manifests mainly with inflammation of the joints or the spine, characterized by pain and stiffness, but IA can also affect other connective tissues, e.g. eyes and skin, and the inflammation can result in irreversible damage of joints and lead to many comorbidities, e.g. cardiovascular disease and osteoporosis [4, 8, 9]. Therefore, a diagnosis of IA can have a substantial impact on an individual’s life and can affect several aspects of quality of life [10]. Fortunately, pharmacological treatment has improved significantly since the nineties, and especially, when treated early, remission is possible. However, management of IA can be complex due to its fluctuating nature, and even in remission, patients experience symptoms such as pain, joint stiffness, fatigue, sleep disturbances, and disability [4, 11, 12].

Studies have shown that especially when newly diagnosed, patients request regular consultations and available support—preferably within the first six months [13–21], as they are particularly fragile and insecure in their new situation. This indicates a particular need for guidance during the period right after diagnosis to enhance emotional, social, and physiological disease management [22]. Increased self-management can improve quality of life in patients with chronic illness [23–29]. Self-management can be defined as: *the individual's ability to manage symptoms, treatments, lifestyle changes, and psychosocial and cultural consequences of illness* [30]. According to the Corbin and Strauss and Lorig and Holman framework [31, 32], self-management programs must include medical management (such as taking medications and attending medical appointments), role management (such as adapting lifestyle and social relations), and emotional management (including processing emotions that arise from having a chronic illness). Furthermore, Lorig and Holman [31, 32] proposed that problem solving, decision making, resource utilization, forming a patient/health care provider partnership and action planning – also called the five core self-management strategies—should be integrated in self-management interventions. Moreover, self-management interventions should include various theoretical perspectives [31, 33–36].

However, when reviewing the substantial number of systematic reviews of arthritis-specific self-management interventions, we found that the effects of interventions are generally small [22, 24, 25, 37, 38] and that comparability of included studies is difficult due to heterogeneity in study design, as interventions are based on different theories, different program focus and modalities and uses different outcomes.

Despite the well-documented need for patient guidance during the period right after diagnosis [22], only a few studies of self-management interventions in chronic conditions have a special focus on the newly diagnosed, and to our knowledge, only one study has, targeted patients newly diagnosed with IA, and here the evaluation was simply based on qualitative data [39].

Therefore, there is a need for additional knowledge about the development of a self-management intervention specifically for this group of patients. New interventions must be systematically developed and evaluated based on qualitative and quantitative methods, in order to learn more about intervention acceptance and fidelity. Also, there is a need to learn more about the mechanisms of impact, efficacy vs. effect, and socioeconomic aspects of these multi-component interventions to make sustainable interventions in the future [40–42]. The Medical Research Council (MRC) provides a framework for developing and evaluating these complex interventions.

### Aim

Therefore, the aim of this study was to develop a self-management intervention targeting newly diagnosed patients with IA, following the MRC Framework.

## Methods

### Design

This study is guided by the UK Medical Research Council’s (MRC) Framework for developing and evaluating complex interventions [40, 41]. This framework divides complex intervention research into four phases: 1) development or identification of the intervention, 2) feasibility, 3) evaluation (randomized controlled trial), and 4) implementation [41]. Here, we solely focus on the development phase.

The entire development and evaluation process should be understood as an iterative process, which is not necessarily sequential [41]; thus, we expect adjustments of the intervention based on the results of the subsequent feasibility test.

### Setting

The intervention was developed at the Center for Rheumatology and Spine Diseases, Rigshospitalet, Denmark. We wished to develop an intervention suitable for this setting and á priori included the interdisciplinary staff employed in this clinic in the development phase. The purpose of this was to gain inputs and perspectives from the interdisciplinary staff and to create ownership, acceptance, and fidelity at all levels, in order to develop a realistic intervention relevant for daily clinical practice. We assume that the involvement of staff will smoothen the transition between feasibility, RCT, and finally, a possible implementation of the intervention in routine rheumatology care.

### Patient involvement

Initially, three patients were involved in the design of the project, including the development of the preliminary intervention. Also, we involved a patient with RA as research partner in the project group in all project phases. Studies have shown that this helps maintain the patient perspective, the relevance of the project focus and structure, and the results [43].

### Overview of the complex intervention development process

**Fig. 1:**
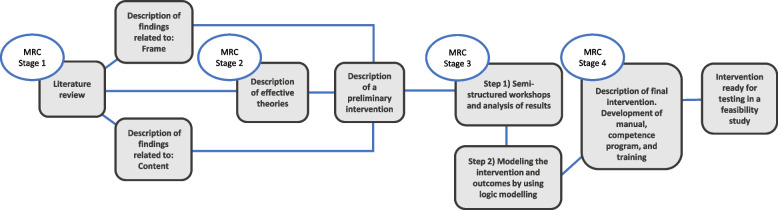
Intervention development stage overview

The stages in the development phase according to MRC are: 1) identifying the evidence base, 2) identifying the theoretical basis for the intervention, 3) modelling process and outcomes, and finally 4) a description of all components and outcomes of the intervention (Fig. 1) [40, 41]. In the following, each of these four phases is described in accordance with the checklist: Guidance for reporting intervention development studies in health research (GUIDED) [44].

#### MRC stage 1. Identifying the evidence base—literature review

**Fig. 2:**
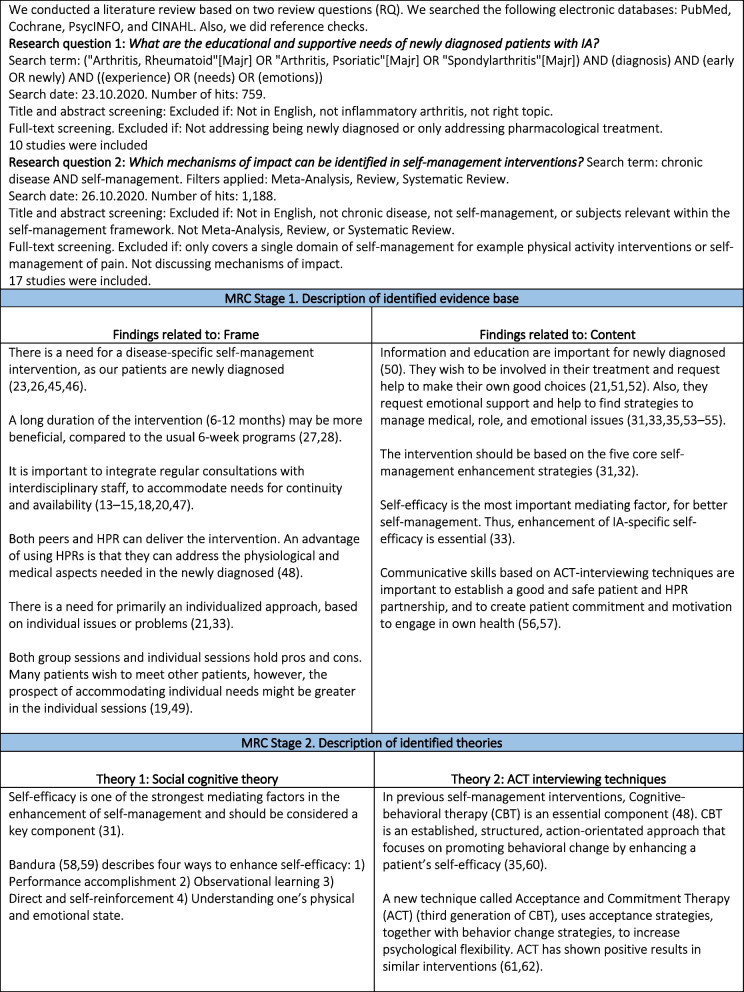
Results from review of the literature [45–62]

According to the MRC, the intervention can be developed either by developing a new intervention or adapting an existing intervention for a new context, based on research evidence and theory [40, 41]. Therefore, we conducted a literature review based on two research questions to support the development of the intervention and to assess if existing interventions were suitable for our patient’s needs and our context or if we needed to develop a new intervention (Fig. 2).

The research team (LHL, TT, AdT, MAa, MLH, SDK, BAE) discussed key results from the review. First, we explored educational needs and supportive needs of the newly diagnosed. Next, we discussed the mix of intervention components regarding successful self-management interventions in patients with chronic conditions in general. Finally, we considered which results were appropriate to our setting.

The results of the literature review related to the frame and content based on the two research questions can be seen in Fig. 2. Further details of the literature reviews are presented in the supplementary material Tables A and B.

#### MRC stage 2. Identifying theory

Evidence shows that using theories in research will increase the quality and effectiveness of health interventions, making a theory-based intervention more likely to be effective than a purely empirical or pragmatic approach [63]. Therefore, we sought to identify a theoretical framework that could help us identify the essential elements in our self-management intervention [64].

During the literature reviews, we came across several theories that have been used in previous self-management interventions. We reviewed psychological theories of behavior change that incorporated the constructs of interest. Given the fact that self-management is built upon Social Cognitive Theory [58, 65], this was chosen as the underlying theory along with Acceptance and Commitment Therapy (ACT) [66] to support the enhancement of self-efficacy (Fig. 2).

### Description of a preliminary self-management intervention

Based on stages 1 and 2, a preliminary intervention was described and discussed with two of the involved patients.

The preliminary intervention drafted by BAE consisted of a combination of individual consultations and group sessions embedded in a multidisciplinary team of nurses, physiotherapists (PT), and occupational therapists (OT). The sessions would focus on e.g., living with a chronic disease, knowledge about IA, unwrapping actual challenges and how to manage dominating symptoms, emotional distress, medical treatment as well as maintenance of a physical and socially active life. Details of the entire development phase are presented in Supplementary material in Tables A and B.

A description of the preliminary intervention was used as inspiration for the workshop discussions.

#### MRC stage 3. Modeling process and outcome

The possibility of reproducing a complex intervention is related to how explicitly the mechanisms of impact and its theory are specified. Thus, modeling a complex intervention can illustrate the underlying premises that are included in the intervention [67]. In our study, the modeling process covered two steps 1) Semi-structured workshops and analysis of results and 2) Modeling the intervention and outcomes [68]:

##### Step 1) Semi-structured workshops and analysis of results

For details about aim, methods, analysis, and results, see Table 1.

**Table 1:** The semi-structured workshops

**Aim**: To obtain inspiration to complete the development of the complex self-management, and to ensure that the intervention was in accordance with newly diagnosed patients’ needs, based on available evidence and clinicians’ experience. Also, that the intervention would be realistic and feasible in clinical practice.
**Method**: Initially, we planned three large workshops, but, due to the COVID-19 pandemic (2020-), we conducted seven smaller workshops. Here, consensus work was conducted with HPRs who had experience in providing care for patients with IA. The semi-structured workshops covered the following topics: a) discussions of the preliminary intervention b) feedback on HPR competencies needed and c) a brainstorming regarding possible outcomes.
**Recruitment:** The recruitment of participants for the various workshops was based on purposive sampling. We contacted relevant clinical managers who were asked to identify doctors and nurses of both genders with a variety of experiences in rheumatology. We recruited participants for the primary sector workshop by contacting the management of a few municipalities. Patients for participation in the workshops were recruited from our user panel.
**Workshop attendees**: Two patients (eight were invited but cancelled because of the Covid-19 pandemic), three occupational therapists, six physiotherapists, 10 medical doctors, one psychologist, one social worker, and 17 registered rheumatology nurses (RRN). We required a minimum of two years of rheumatology experience.
**Conducting workshops:** In advance: Information material was sent to participants in the workshops to prepare them for the discussions. The information material included a short description of the theoretical framework of self-management On the day: The initial discussion concerned the newly diagnosed patients’ characteristics and their concerns and needs. This was followed by a discussion of the preliminary intervention and of HPR’s need for competence development. Finally, the participants were asked which changes they would like to see in the patients as a result of the intervention. Every topic for discussion began with a short introduction and a few minutes for individual reflection and note-taking. Thereafter, the discussions were guided by open-ended questions and in-depth questions by an experienced moderator (BAE) Example of questions: Which existing treatments and offers (in addition to the pharmacological one) can support the newly diagnosed? Which psychosocial problems do newly diagnosed patients experience? Which challenges are present when providing/receiving support? What could be the best set-up for providing/receiving support? (Interview guide is available in supplementary material Table C).
**Data collection and analysis:** The workshops were audio-recorded after taking informed consent. All data were collected and held in accordance with data protection guidelines. All data material was transcribed, coded, and categorized using the data management system NVIVO 12. Data was analyzed together with notes from the participants using a thematic analysis approach inspired by Braun and Clarke’s six-step method [69].
**Results from the workshops**: Topic a) Discussions of the preliminary intervention. In the workshop, several components in the preliminary intervention were confirmed as relevant and new components arose. Enhancement of self-efficacy in face-to-face sessions with a focus on personal interaction and dialogue based on personal challenges was confirmed along with expectation alignment about treatment and consequences of the diagnosis. Time was a recurring theme in the workshops but in different contexts. Sufficient time for conversation in each session was highlighted as well as the duration of the complete intervention. An intervention more than six months was preferred. The element in the frame of the intervention that caused the most discussion was whether the intervention should include group sessions. Both patients and HPRs mentioned that patients need to talk to others, preferably someone who has had the disease for a longer time. Some also argued that the newly diagnosed were not ready for group sessions as they were too emotionally distressed. The participants also discussed if the groups should be stratified by gender, age, ethnicity, level of education, and symptoms, or if they should be thoroughly mixed. Acceptance, crisis, hope, and existential issues were not very explicitly described in the preliminary intervention. However, these emotional reactions were mentioned several times in the workshops. Especially the patients expressed that acceptance is a prerequisite for a better quality of life (Results are presented in supplementary material, Tables A and B). Topic b) Feedback on HPRs competences HPRs delivering the intervention should hold the following competencies: knowledge in medical, social, and emotional disease management, as well as competencies in communication and questioning techniques and how to be a good facilitator. Topic c) Brainstorm regarding possible outcomes The outcomes suggested in the workshops were: compliance, fatigue, pain, morning stiffness, physical function, health-related quality of life, anxiety, and depression, workability, physical activity, health belief, illness perception, and self-efficacy.

##### Step 2) Modeling the intervention and outcomes by using the logic modeling

**Fig. 3:**
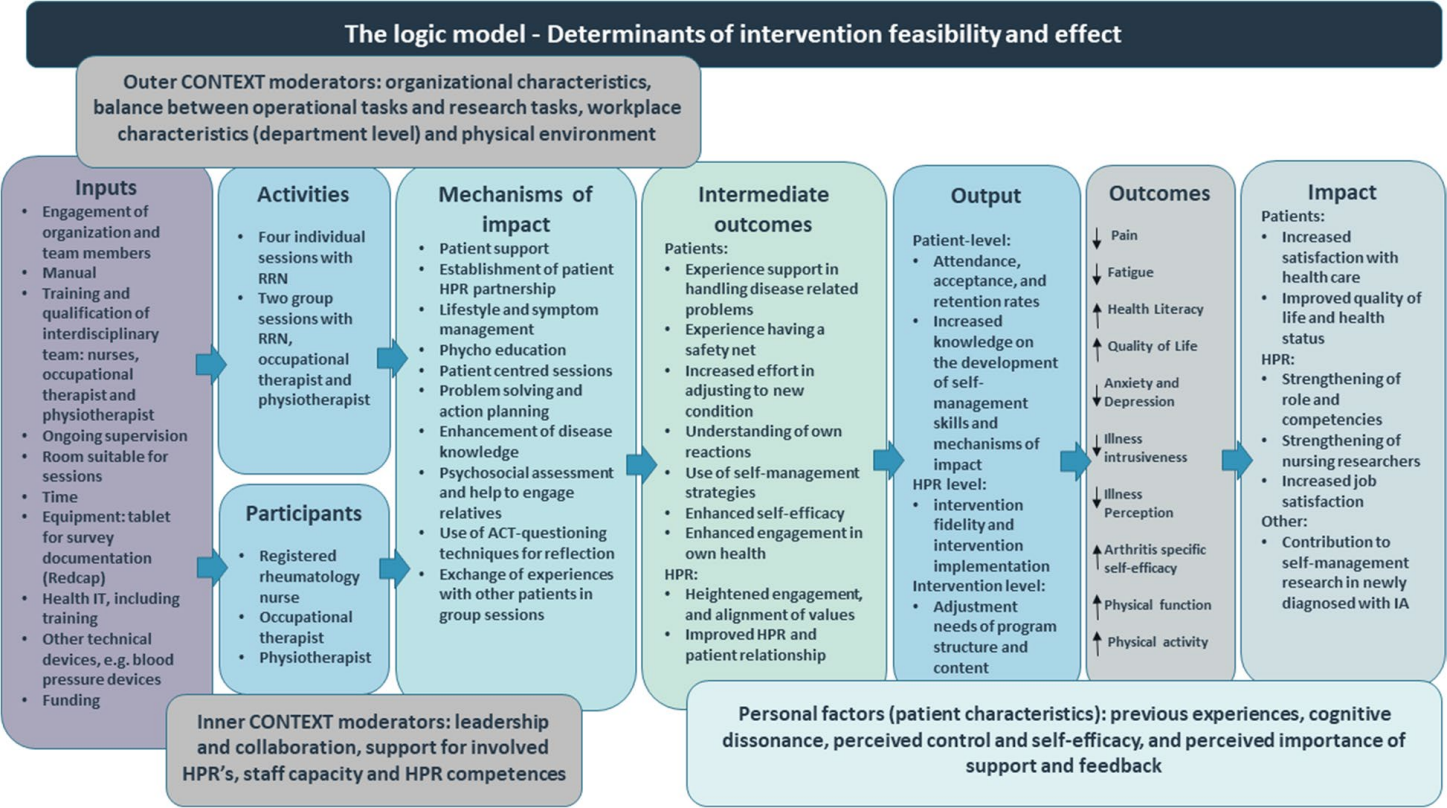
The logic model RNN: Registered Rheumatology Nurse; ACT: Acceptance and Commitment Therapy; HPR: Health Professional

In line with the MRC guidance, we developed a logic model to present the theoretical underpinning of the intervention [42]. See Fig. 3 for details.

This provided an overview of the assumed mechanisms in the intervention and how the theory and assumptions underline the intervention. The logic model was refined throughout the intervention development process. The model covers eight core elements [70, 71]: Inputs (available necessary resources), Activities and Participants (activities in the intervention and who is delivering the intervention), Mechanisms of change (the expected behavior change mechanisms), Intermediate outcomes (the immediate benefits), Output (process evaluation measures), Outcomes (the direct benefits for the patients), and Impact (the long term endpoints).

The final model integrated results from the literature review, the chosen theory, workshops, and other feedback.

### Selection of outcomes

Unfortunately, there is no validated single measure of self-management. Self-management is a complex construction with a person-centered approach, that addresses medical, social, and emotional issues, and historically the effect of self-management interventions have been measured with a great number of outcomes [38]. Systematic reviews [22, 24, 25, 37] have found that comparability of included studies was difficult, as they found over 70 variables, interventions were poorly described and data were collected with different measurement instruments. The results from the reviews showed a marginal effect of arthritis self-management interventions, perhaps because studies frequently assess outcomes that are not particularly targeted in these interventions. E.g., measuring pain, where the aim is not a reduction in pain, but a reduction in the perception of pain [22, 24, 25, 37]. Thus, it is relevant to discuss, what to measure, why the effects are sparse, and which outcomes these interventions address [22]. A recently published systematic self-management outcome review [38] identified all patient-reported outcomes (PROs) and validated questionnaires used to measure self-management. Together with responses from the workshops and the literature, we chose relevant outcomes (Table 2). Subsequently, we identified relevant validated questionnaires to measure our selected outcomes. These questionnaires have previously been validated in a similar population and can be used in a clinical setting to investigate the effect in randomized controlled trials.

**Table 2:** Outcomes and measurements

Outcome	Patient-reported outcome measurements (PROMs)
Pain intensity	The Visual Analogue Scale (VAS) [72, 73]
Fatigue	Bristol Rheumatoid Arthritis Fatigue Questionnaire (BRAF) [74]
Health literacy	The Health Literacy Questionnaire (HLQ) [75]
Quality of life	EuroQol-5 Domain (EQ5D) [76]
Anxiety and depression	The Hospital Anxiety and Depression Scale (HADS) [77]
Illness intrusiveness	Illness Intrusiveness Rating Scale (IIRS) [78]
Illness perception	The Brief Illness Perception Questionnaire (IPQ) [79]
Self-efficacy	The Arthritis specific self-efficacy measurement tool (ASES) [80]
Physical function	The Modified Health Assessment Questionnaire (MHAQ) [81]
Physical activity and sedentary time	The Physical Activity and Sedentary Time Questionnaire (FAST) [82]

We carefully considered the order of the demographic questions and the questionnaires, and our collection of validated questionnaires was face-validated by five patients through cognitive interviews [83] with rheumatoid arthritis or psoriatic arthritis (23–77 years/ three men and two women). The respondents were selected to ensure equal distribution across age and gender. Adaption of the order of the demographic questions and the questionnaires was made accordingly.

#### MRC stage 4. The final intervention

Through the two-step modeling process, the final intervention NISMA (Newly diagnosed with Inflammatory arthritis – a Self-MAnagement intervention) was developed. The research team agreed on of mix of individual and group sessions. The same rheumatology-trained nurse should facilitate all the four individual sessions. To demonstrate interdisciplinary agreement, the group sessions consisted of a nurse, an occupational therapist (OT), and a physiotherapist (PT), with the nurse being the facilitator.

The workshops uncovered a need for a smaller time range between the sessions at the beginning of the intervention, and we decided to allocate the sessions as illustrated in Table 3.

**Table 3:** The NISMA intervention

Session type	Topic	Timeline	Duration	Aim	Activity
**First individual session with a nurse**	Medical management	14 days after baseline	1.5 h	To establish a partnership between patient and nurse, based on patient values, and health history. And to increase knowledge about self-management and physical state	Introduction to self-management and the frame of the sessions What is arthritis – education and information about principles of medical treatment Management of individual problems – help to achieve performance accomplishment through problem-solving and action planning
**Second individual session with the nurse**	Emotional management	6 weeks after baseline	1 h	To increase knowledge about emotional reactions	Normal emotional reactions including acceptance and crisis theory Management of individual problems – help to achieve performance accomplishment through problem-solving and action planning
**Third individual session with a nurse**	Role management	3 months after baseline	1 h	To help prioritize and address challenges related to work and social life	Social relationships, identity, and loneliness. The outside world's expectations versus own expectations Management of individual problems – help to achieve performance accomplishment through problem-solving and action planning
**First group session with a nurse, occupational- and physiotherapist**	Symptom management	4 months after baseline	2 h	To help patients meet other patients and make a room for observational learning about symptom management	Symptoms as flare, pain, fatigue, and sleep problems. Symptom interactions Each patient tells a little about themselves and shares their experiences with IA and IA-symptoms
**Second group session with a nurse, occupational- and physiotherapist**	Lifestyle and co-morbidity	6 months after baseline	2 h	To help patients meet other patients and make a room for observational learning in relation to lifestyle management	Each participant shares their experiences since the last session Lifestyle. What is good and what is bad—why? Identify the need for lifestyle changes and possible barriers and solutions. Patients share previous experiences with lifestyle changes Management of individual problems – problem-solving and action planning
**Fourth individual session with a nurse**	Future use of healthcare system and round up	9 months after baseline	1 h	To increase health literacy	Use of healthcare and future collaboration with healthcare professionals Management of individual problems – problem-solving and action planning

### Manual, HPR competence development and training

The research team developed a comprehensive manual, describing each session and the overall intervention strategy and framework. Our patient research partner and experts in rheumatology and self-management commented on the content to secure content validity. In addition, we conducted cognitive interviews with the HPRs to determine the face validity of the manual and we conducted a two-day competence program in October 2021 to train HPRs in delivering the intervention to secure fidelity and acceptance.

## Discussion

In this paper, we have described the development of a complex self-management intervention aimed at increasing self-management in patients newly diagnosed with IA. Throughout the development process, we followed the MRC framework for the development of complex interventions [41]. This approach has made the development phase dynamic, systematic, feasible, and transparent.

According to applicable EULAR (European Alliance of Associations for Rheumatology) recommendations [84], self-management should be included in daily rheumatology care to support patients to become active partners in their treatment. EULAR highlights the importance of including patient education and key self-management interventions such as problem-solving and action planning as well as a CBT approach in rheumatology practice. All these elements have been included in our intervention. From the literature reviews, we identified what we believe to be active ingredients in effective self-management interventions including theoretical underpinning and rationale for behavior change, as illustrated in our logic model. The intervention was adjusted to our setting and our population through workshops. We believe that the identified components are essential to increase self-management in newly diagnosed patients with IA. However, the optimum mix of intervention components in the self-management of newly diagnosed patients with IA remains uncertain, and it is still unknown whether a subtle change in the components, mode, or intensity of our self-management intervention can optimize outcomes. In addition, little is known about how to distinguish attenders from non-attenders in self-management interventions for chronic diseases [29].

Several patient-related factors in these types of interventions influence the effects of self-management interventions. These include demographic factors such as socioeconomic status and culture, clinical factors such as comorbidities and complexity of the treatment regimen, and system factors such as quality of relationships and communication with HPRs [85]. Large variation in effect size between patients has been demonstrated in systematic reviews [29, 49]. So not only do we need to identify ‘what works best?’, but we also need to identify ‘what works best for whom?’ and to adjust the content in the intervention to the individual level. We hope that we with our individually tailored and person-centered approach will be able to accommodate this need. However, this also leaves us with a black box, as the person-centered approach makes it difficult to pinpoint exactly how the intervention was delivered and what the content was. Therefore, a thorough process evaluation will be conducted in our feasibility study, to evaluate both context, content, and individual factors [42].

Through the workshops, we involved all levels of the organization, both to optimize the intervention, but also to create ownership and commitment and to identify barriers and shortcomings of the preliminary intervention. As a result of the workshop, we identified potential problem areas to which we will pay particular attention in the feasibility study. These areas include capacity in the clinic, time allocated for conversation, continuity in and duration of the intervention, and stratification in group sessions. We have involved patients and HPRs in the identification of relevant outcomes and the identification of HPR's competence upgrading needs.

This project can only be successfully conducted if the HPRs understand the principles of self-management and the operational theories are used. To create fidelity and acceptance of the intervention, we have prepared a comprehensive manual, a competence development program, and continuous supervision for the HPRs. In addition, intervention acceptance and fidelity will be explored in the feasibility study from both the patients’ and the HPR’s perspectives through observations and interviews. Such strategy is in accordance with the MRC framework which highlights that process evaluation is essential to designing and testing complex interventions [41, 42].

With this intervention, we aim to strengthen rheumatological nursing and multidisciplinary collaboration. In addition, we hope that supporting patients successfully self-manage their arthritis, can improve their quality of life and prevent unsustainable health care costs in the future. 

### Strengths and limitations

Our systematic approach based on the MRC framework [40, 41] has secured a transparent and rigorous process. The NISMA intervention was based on current evidence and further adapted in close collaboration with patients, HPRs, rheumatologists, the research team, and the clinic’s management team.

Because of the Covid pandemic, only two patients attended the workshops, which is a limitation, as more patients could have given a broader perspective. However, the workshop data, systematic review, and collaboration with patient partners ensured that the intervention design included a solid patient focus.

## Conclusion

NISMA—A nurse-led complex self-management intervention embedded in a multidisciplinary team, has been developed and described based on MRC’s framework for the development of complex interventions. The intervention is targeted at increasing self-management in the newly diagnosed and consists of several components to accommodate the complex issues the newly diagnosed may have. The intervention is currently being tested in a feasibility study.

## Supplementary Information

**Additional file 1: Table A.** Intervention development – frame [86–94].

**Additional file 2: Table B.** Intervention development – content.

**Additional file 3: Table C.** Workshop Interview guide.

## Data Availability

The datasets supporting the conclusions of this article are included within the article and in the supplementary material.
