# Supplementary material for: Newly diagnosed with inflammatory arthritis (NISMA)–development of a complex self-management intervention
Source: BMC Health Serv Res. 2023 Feb 7;23:123. doi: 10.1186/s12913-022-09007-w (PMC9902823; doi:10.1186/s12913-022-09007-w)
Supplement: Supplementary file 1 — Additional file 1: Table A. Intervention development – frame [86–94]. [file 12913_2022_9007_MOESM1_ESM.docx]

**table A. Intervention development – frame**

| **Intervention component** | **Results from the literature reviews** | **Preliminary intervention** | **Workshop results** | **Final Intervention** |
| --- | --- | --- | --- | --- |
| **Theoretical framework** | Self-management interventions should be based on a theoretical framework. Interventions based on SCT or CBT were more effective.  Bandura (59,65) describes four ways to enhance self-efficacy: 1) Performance accomplishment 2) Observational learning 3) Direct and self-reinforcement 4) Understanding one’s physical and emotional state.  An additional influence on the development of self-management interventions has come from clinical psychology, particularly cognitive-behavioral therapies (CBT)(48). A new technique called ACT has shown promising results and is suitable for our setting (61,62). | We chose our theoretical frame to be SCT with components of CBT, as we assessed that self-efficacy enhancement strategies were very important in the newly diagnosed. | Self-efficacy was mentioned but not directly addressed. Patients need help to learn to listen to their bodies and gain confidence in one’s abilities. Professional support provides security. HPR should help the patients take responsibility for the situation and teach them to control their arthritis. | Social cognitive theory and enhancement of self-efficacy have a great focus in the final intervention. Core basic self-management enhancement strategies will be addressed in all sessions.  We will use a personal approach based on ACT interviewing to support interviewing techniques. |
| **Generic or disease-specific** | Disease-specific self-management interventions are reported to produce better outcomes compared to generalized chronic disease self-management interventions, and it is thought that this approach may be more beneficial for patients with IA (23,26,45,46). | IA specific | It was confirmed that addressing arthritis-specific mechanisms are of great importance in the newly diagnosed. | IA is specific with education in pathological mechanisms, medication, and general symptoms. |
| **Duration in months** | Most of the tested interventions have a duration of 6-8 weeks or less, and many of them only produce short-term significant improvements (28,86). A review of self-management interventions in patients with heart failure and patients with COPD, also revealed that interventions with a longer duration (in months) could lead to better health outcomes (29), and a reduction in health disparity has also been seen in longer interventions (6–12 months) that were individually tailored (49). | 6-12 months. | Duration more than 6 months was mentioned. The results showed that because the newly diagnosed are in an overwhelming situation, there is a risk of information overload.  Time between sessions to process feelings and emotions I needed. Thus, time is both a ‘helpful’ factor and term in the coping process, and some process faster than others. | To minimize the timespans between the sessions we decided to intervene for nine months, with a shorter period between sessions at the beginning of the intervention.  As the intervention had to fit the current setting, manageable for the patients to attend we did not wish to increase the number of sessions. |
| **Continuity and availability** | Qualitative studies (13–18,20) have shown that patients request regular consultations and available support with HPRs especially when newly diagnosed. Furthermore, a good relationship positively influences self‐efficacy, which results in increased adherence behaviors and a better acceptance of the diagnosis (87). | 3 individual and 3 group consultations and the possibility to call the outpatient clinic. | The need for regular consultations and availability from HPR was confirmed. In addition, HPR needs to show availability and keep close contact during the first year. | Four individual sessions and two group-session were distributed over nine months. Regular consultations and the possibility to call the outpatient clinic. |
| **Peer or HPR delivered** | Many self-management interventions are delivered by peers, under the rationale that people who themselves have a chronic disease are better at teaching others how to self-manage (88), and the evidence does suggest that lay leaders can teach arthritis self-management courses with results similar to those achieved by professionals (89). However, as we are in a hospital setting, and our population is newly diagnosed with a need for disease-specific knowledge, a health professional seemed most appropriate. It could be considered to use a peer as a ‘co-supervisor’ in some of the sessions (48). | Due to the medical aspects of IA around mechanisms, medication, and IA classification a nurse was deemed most appropriate in close cooperation with other HPRs | The importance of an interdisciplinary intervention was confirmed. Both because patients need help from different types of HPRs, and messages are reinforced when HPRs confirm each other.  Such an approach provides a feeling of professional security in patients. | The nurse delivered the intervention in corporation with occupational and physiotherapists in the group sessions. |
| **Group and/or individual sessions** | Most self-management interventions are group-based, however, both individual and group sessions hold different pros and cons. Group sessions have been found to produce higher attrition rates and disparity in vulnerable groups, and high rates of dropout from group programs have been reported in several reviews of chronic disease interventions in low socioeconomic status and other vulnerable groups, while other reviews have noted that individually tailored interventions appear to reduce disparity (49). As it is easier to individualize content in individual sessions, many patients prefer one-to-one discussions (19). | Three individual sessions with a specially trained nurse.  Three group sessions (6-10 participants in each group) supervised by an RRN, a physiotherapist (PT), or an occupational therapist (OT). | All agreed that individual session was beneficial. We found conflicting results regarding the group sessions.  Arguments for group sessions: the patients wished to meet other patients with IA to exchange experiences, and to decrease loneliness.  Arguments against: Groups cannot meet individual needs. Most agreed that immediately after diagnoses most patients would be too vulnerable to attend group sessions and that the groups were too large. | The number of individual sessions was increased from three to four sessions.  The group size was decreased to 5 persons and the number of sessions was decreased to two sessions,  Because of the wide agreement concerning newly diagnosed not being ready for group sessions, the group sessions were placed after 4 months and 6 months. |
| **Duration of each session** | No evidence was found. | Individual sessions: duration 30 minutes.  Group sessions: duration 1 hour. | It was discussed if 30 minutes in the individual sessions and 1 hour in the group sessions were sufficient time. Most agreed that it was not. | Duration of individual sessions: increased from 30 minutes to 1.5 hours in the first session and 1 hour in the remaining session.  Duration in the group sessions was increased to 2 hours. |
| **Face to face or online sessions** | There is evidence supporting the effectiveness of online interventions compared to treatment as usual when combined with personal contact, email, or telephone support (90–92). However, a personal relationship is supported by face-to-face consultations, and a good relationship is perceived as being important for the feeling of confidence and well-being (93). | Face-to-face sessions were chosen for this setting, as close personal interaction and communication are assessed as beneficial for the HPR-patient relationship. | Close contact is important, and much communication is lost in online sessions; however, it might increase attendance. | The preferred setting is face-to-face sessions, however, if the patients have a great wish for online sessions this is possible. |
