# Supplementary material for: Newly diagnosed with inflammatory arthritis (NISMA)–development of a complex self-management intervention
Source: BMC Health Serv Res. 2023 Feb 7;23:123. doi: 10.1186/s12913-022-09007-w (PMC9902823; doi:10.1186/s12913-022-09007-w)
Supplement: Supplementary file 2 — Additional file 2: Table B. Intervention development – content. [file 12913_2022_9007_MOESM2_ESM.docx]

**Table B. Intervention development – content**

| **Intervention Content** | **Results from the literature reviews** | **Preliminary intervention** | **Workshop results** | **Final Intervention** |
| --- | --- | --- | --- | --- |
| **Individualized content** | The intervention should be individually tailored with the opportunity to shape the content according to personal needs. Patients wish to be involved in their treatment and request help to make their own good choices (21,51,52) Involving the patient in the decision-making is positively associated with the patient’s sense of trust, control, and self-efficacy. It was negatively associated with patient feelings of uncertainty (94). | The sessions will focus on patients’ actual challenges in living with IA “everyday life” both emotional, social, and physical. And will integrate training in specific tools to solve challenges. | An individual approach with time to listen and help the patient prioritize time and activities was highlighted.  Goals should be adjusted according to patient readiness. | The first three individual sessions will be individually tailored with the opportunity to shape the content according to personal needs. Every session has an educational topic. However, personal issues and needs are highly prioritized. Also, the group sessions have overall topics, with build in time for discussions of personal issues. |
| **Hold more components** | Evidence indicates that effective self-management interventions should contain more components and carry a large educational component.  To attain self-management skills, patients must be exposed to several behavior change strategies targeting medical, role, and emotional challenges (31,33–35). This also includes symptom management and lifestyle management (51,53,54). | Both types of sessions will focus on e.g. living with a chronic disease, knowledge about IA, unwrapping actual challenges and how to manage dominating symptoms, medical treatment as well as maintenance of a physical and socially active life. | The following themes were mentioned: Altered family roles, and the influence on relatives, along with loneliness and altered family roles. Concerns about work-life, economy, and livelihood. The shock of having been diagnosed, and thoughts and worries about the future. The expectation of getting ’old’ life back versus the consequences of the diagnosis. Acceptance is a prerequisite for a better quality of life - a process that takes time. Give hope for a good life. | The first three individual sessions will address medical, emotional, and role management strategies. Also, physical and emotional reactions to diagnosis and symptoms, including crisis theory and symptoms of depression.  Content in the group sessions: will address lifestyle and risk of comorbidity and symptom management, including sleep disturbances, fatigue, pain, and flare. |
| **Enhancement strategies** | Lorig and Holman proposed five core self-management strategies that should be addressed in self-management interventions (31,32): 1) problem solving, 2) decision making, 3) resource utilization, 4) forming of a patient/health care provider partnership, and 5) taking action (31). Self-efficacy can be promoted through social modeling - own and observed experiences, mastery - you reach your goal, encouragement from others (persuasion), and help to understand the physical and emotional state and understand the consequences of a given behavior (58,65). Approaches should be underpinned using core communication skills to build trust in the patient-provider relationship (56,57). | Educational components, as the patients are newly diagnosed, and elements of social cognitive theory, motivational interviewing, cognitive-behavioral theory together with core self-management theories. As goal setting is a key component, it will be used, if it fits the nature of the problem, and the patent is interested in using this approach. | The nurse must be able to guide the patient through questioning techniques.  Communication at an individual level and adjusting goals according to patient readiness.  Answers should not necessarily provide a solution but contribute to increased reflection.  Patients need help to find out what is important and help to prioritize. | Include core self-management strategies including enhancement of self-efficacy. guided by ACT interviewing techniques.  As a result of the workshops, we have adjusted and added a special focus on acceptance and crisis theory as this is a population of newly diagnosed patients. Self-efficacy theory confirms that getting to know one’s emotional response increases self-efficacy, thus addressing the natural grief end crisis response seems appropriate, as it both increases self-efficacy and acceptance. Thus, understanding illness response is a prerequisite for acceptance of illness. |
