# Supplementary material for: Newly diagnosed with inflammatory arthritis (NISMA)–development of a complex self-management intervention
Source: BMC Health Serv Res. 2023 Feb 7;23:123. doi: 10.1186/s12913-022-09007-w (PMC9902823; doi:10.1186/s12913-022-09007-w)
Supplement: Supplementary file 3 — Additional file 3: Table C. Workshop Interview guide. [file 12913_2022_9007_MOESM3_ESM.docx]

**Table C. Workshop Interview guide**

| **INTERVIEWGUIDE WORKSHOPS** | |
| --- | --- |
| **PART 1. The newly diagnosed (30 minutes discussion)** | |
| **SUBJECT** | **QUESTIONS** |
| What works? | - Please describe which physical symptoms the newly diagnosed patients have. - Please describe which psycho-social problems the patients have. - Please mention which existing treatments (besides the medical one) helps the newly diagnosed patients with IA. |
| What is challenging? | - Please describe the challenges you experience when you support newly diagnosed patients with IA in clinical practice. |
| **PART 2. The Intervention (30 minutes discussion)** | |
| How and what can be improved? | - What is your response to the mix of individual and group sessions? - What should the focus be in the sessions? - What are your considerations about the group sessions in relation to gender, diagnosis, and age? - What should the effect (outcomes) be of implementing this intervention? - Which outcomes/changes would you expect to identify in the patients? - What resources will it require? - What is realistic? - Which competencies are needed in the HPRs to deliver this suggested intervention? - Think of the intervention from a contemporary perspective – including during COVID-19. What would it require? |
